# The Rapid Growth of Fibroids during Early Pregnancy

**DOI:** 10.1371/journal.pone.0085933

**Published:** 2014-01-20

**Authors:** Laura Benaglia, Lucia Cardellicchio, Francesca Filippi, Alessio Paffoni, Paolo Vercellini, Edgardo Somigliana, Luigi Fedele

**Affiliations:** 1 Obstet-Gynecol Dept, Fondazione Ca’ Granda, Ospedale Maggiore Policlinico, Milan, Italy; 2 Università degli Studi di Milano, Milan, Italy; University of Edinburgh, United Kingdom

## Abstract

Several studies aimed to disentangle whether pregnancy influences the growth of uterine fibroids but results were inconsistent. In this study, we speculated that fibroid enlargement during pregnancy may not be linear and we hypothesized that this phenomenon may mainly occur during initial pregnancy. To test this hypothesis, we set up a prospective cohort study of women with fibroids undergoing IVF. Cases were women achieving a viable pregnancy. Controls were the subsequent women with fibroids but failing to become pregnant. Twenty-five cases and 25 controls were recruited. The total number of fibroids in the two groups was 46 and 41, respectively. The mean ± SD diameter of the fibroids was 17±10 and 20±11 mm, respectively (p = 0.18). A statistically significant enlargement emerged exclusively in pregnant women. The median (Interquartile Range) modification of the diameter of the lesions in cases and controls was +34% (+6%/+65%) and +2% (−6%/+12%), respectively (p<0.001). The median (Interquartile Range) modification of the volume of the lesions was +140% (+23%/+357%) and 0% (−18%/+37%), respectively (p<0.001). In pregnant women, we failed to document any significant correlation between the magnitude of the growth and ovarian responsiveness to hyper-stimulation, suggesting that steroids hormones are not the unique factors involved. In conclusion, fibroids undergo a rapid and remarkable growth during initial pregnancy. Reasons behind this phenomenon remain to be clarified. The early rise in steroids hormones during early pregnancy may not be sufficient to explain the process. Other pregnancy-related hormones and proteins may play also key roles.

## Introduction

The influence of pregnancy on uterine fibroids is debated for up to three decades. Several studies aimed to disentangle whether pregnancy determine the growth of these lesions but evidence is inconsistent and a firm conclusion has yet to be reached [1]–[7]. Differences in study designs and population studied may explain these discrepancies. Of particular relevance here is the time points of the ultrasound assessments since the pattern of fibroid growth during pregnancy may not be linear.

In this study, we hypothesized that fibroid growth may mostly occur during early pregnancy. Few small studies evaluated the impact of initial pregnancy and the results are conflicting [3], [5], [6] (Table 1). To shed more light on this topic, we focussed on women achieving pregnancy through IVF since this condition offered us the unique opportunity to prospectively obtain precise data on the presence and dimension of the fibroids immediately before the advent of pregnancy and to monitor their modification very early during pregnancy. Noteworthy, we recently demonstrated that fibroid growth is not affected by controlled ovarian hyperstimulation for IVF, thus protecting our study design from the theoretical confounding effect of elevated sex steroids hormones [8].

**Table 1:** Studies evaluating fibroids size before and during pregnancy.

Study	N. women(lesions)	Recruitment time	Results
Rosati *et al*., 1992	12 (12)	Winthin 4 months prior to pregnancy	Significant increase in volume (+8%, p<0.001).
Neiger *et al*., 2006	11 (n.r.)	Within 6 months prior to pregnancy	Prepregnant size 88±64% of the pregnant size (p = ns)
Ozturk *et al*., 2009	9 (n.r)	n.r.	No significant modifications but precise data not shown

n.r.: not reported.

## Materials and Methods

Patients who were selected for IVF-ICSI between January 2012 and November 2012 at the Infertility Unit of the Fondazione Ca’ Granda, Ospedale Maggiore Policlinico were considered for study entry. Women were eligible if they were diagnosed at least one uterine fibroid with a mean diameter ≥10 mm prior to enter the cycle. Women carrying more than four myomas were excluded. Patients with submucous fibroids encroaching on the cavity line, those with at least one fibroid with a diameter >50 mm and those with fibroid-related bleeding were also excluded and advised to have myomectomy. Difficult sonographic evaluation of the characteristics of the uterus and the presence of suspected adenomyosis were additional exclusion criteria. Cases were women achieving a viable pregnancy as assessed 4–5 weeks after embryo transfer. Controls were the subsequent women with fibroids performing embryo transfer but failing to become pregnant. Women with early abortion or ectopic pregnancy were excluded. The local Institutional Review Board “Fondazione Ca’ Granda, Ospedale Maggiore Policlinico” (N. 2413) approved the study and all recruited patients signed an informed consent.

Selected patients underwent transvaginal ultrasound between day 1 and day 8 of the cycle during the month preceding the beginning of hyperstimulation. The precise location and dimension of all the visible myomas were recorded at this time. Even if the presence of at least one fibroid with a mean diameter ≥10 mm was an inclusion criterion, concomitant fibroids of smaller size were also measured and studied provided that the total number per woman did not exceed four. Hystero-sonography was done if necessary to rule out submucosal location. Pregnant patients were systematically scanned 4–5 weeks after embryo transfer and were definitely enrolled in the study only if the US scan demonstrated the presence of a viable embryo. The second US assessment of the precise location and dimensions of the fibroids was performed at this time. Controls were matched to cases by study period. They corresponded to the subsequent women with fibroids performing embryo transfer but failing to become pregnant (negative human Chorionic Gonadotropin-hCG assessments at +14 and +16 days after oocytes retrieval). Similarly to cases, they were scheduled a US assessment to evaluate the fibroids 4–5 weeks after the embryo transfer. None of women who failed to achieve pregnancy received estroprogestins or progestins in the period between the embryo transfer and the following second ultrasound assessment. Sonographic appearence of myomas was defined as symmetrical, well-defined, hypoechoic and heterogeneous masses [9]. Three perpendicular diameters (d1, d2, d3) were measured for each myoma. The diameter of the lesions used for the analysis corresponded to the mean of these three diameters. The volume was calculated based on the following formula: 4/3*π*(d1*d2*d3)/8. The precise location of the lesion was recorded in details to allow for paired analyses. A myoma with >50% of its diameter bulging out of the uterine contour line was defined as subserous. Intramural fibroids were those mostly within the uterine shape. Myomas distorting the cavity line were defined as submucosal and, as mentioned earlier, patients with these lesions were excluded from the study. All scans were performed by three experienced physicians engaged for a long time in gynecological ecographies and who were blinded to the prior condition of the women. The localization of every myoma was described in details based on their location within the uterine wall (subserous or intramural) and referring to the three anatomical planes (sagittal, coronal and transverse planes). Moreover, lesions were graphically recorded on a rough sketch and, if deem useful to avoid any mismatching, pictures were taken and stored to solve possible inconsistencies. Preliminary experiments on 10 volunteers with fibroids showed an inter- and intra-observer variability for the measurement of the diameter of the myomas both consistently below 20%. They both rose to 100% when considering the number of lesions.

Patients selected for IVF-ICSI were monitored and managed according to a standardized clinical protocol as reported elsewhere [10]. Briefly, the dose of gonadotropins was determined on an individual basis according to the characteristics of the patients as age, serum hormonal levels and antral follicles count. Patients underwent serial transvaginal ultrasound and hormonal monitoring during hyperstimulation. When three or more leading follicles with a mean diameter >18 mm were visualized, 250 µg of recombinant hCG was administered s.c. Oocyte retrieval was performed transvaginally 36 hours after the hCG injection. Embryo transfer was performed 48–72 hours after the oocyte collection. Luteal phase support with progesterone 200 mg intravaginally twice a day was started on the day of oocytes retrieval and discontinued after 14 days regardless of the result of serum hCG assessment (pregnant and non-pregnant women thus received the same treatment). Cycles were cancelled because of poor or hyper-response. We defined hyper-response as a serum estradiol level greater than 4000 pg/ml and/or more than 20 follicles identified at ultrasound scan before hCG administration. Poor response was defined by the ecographic evidence of fewer than 3 follicles during ovarian hyperstimulation. Serum hCG evaluations to assess pregnancy was performed at +14 and +16 days after oocytes retrieval. Clinical pregnancy was defined as the ultrasonographic demonstration of an intrauterine gestational sac containing a viable embryo 4–5 weeks after embryo transfer.

Data Analysis was performed using the Statistics Package for Social Sciences (SPSS 18.0, Chicago, IL). The diameter of the fibroid before and after treatment was compared using the paired Student *t*-test and data is reported as mean ± SD. The volume of the fibroid before and after treatment was compared using the Wilcoxon paired test and data is reported as median (IQR: Interquartile Range). Comparisons of the baseline characteristics between the two groups were done using the unpaired Student *t*-test, the Mann-Whitney test or the Fisher Exact test, as appropriate. Probability values <0.05 were considered statistically significant. The sample size was calculated setting the type I and type II errors at 0.05 and 0.20 respectively and based on a per patient basis (thus initially assuming that there was only one lesion per patient). We stated as clinically relevant a relative increase in diameter of 30%. Based on an expected mean ± SD diameter of the fibroids in our population of 22±10 mm [10], the total number of patients to be enrolled was of about 50 women (25 cases and 25 controls).

## Results

Twenty-five women with fibroids achieving pregnancy through IVF were selected. Three of them were twin pregnancies. They were matched to 25 women with fibroids failing to become pregnant. Additionally, we monitored fibroid size modification in an eligible woman who became pregnant spontaneously before initiating the treatment cycle. She was carrying one single intramural fibroid. The diameter of the lesion grew from 20 to 31 mm and the volume passed from 3.8 to 15.0 ml. This case was however excluded from the analysis to prevent confounders. Baseline characteristics of the two study groups are shown in Table 2.

**Table 2:** Baseline characteristics of the study groups.

Characteristics	Pregnant women	Non pregnant women	p
	n = 25	n = 25	
Age (years)	36.7±3.4	37.2±3.8	0.61
BMI (Kg/m^2^)	22.5±2.8	23.4±3.8	0.33
Previous pregnancies	9 (36%)	10 (40%)	1.00
Previous myomectomy	2 (8%)	4 (16%)	0.67
Previous IVF cycles	10 (40%)	9 (36%)	1.00
Duration of infertility (months)	57±25	49±23	0.28
Day serum FSH (IU/ml)	7.7±2.2	7.7±3.2	0.95
Serum AMH (ng/ml)	2.3±1.6	1.9±2.2	0.45
Indication to IVF-ICSI			0.11
Male factor	12 (48%)	8 (32%)	
Tubal factor/endometriosis	4 (16%)	6 (24%)	
Unexplained	6 (24%)	2 (8%)	
Mixed	3 (12%)	9 (36%)	
Protocol of ovarian stimulation			1.00
Long protocol	13 (52%)	13 (52%)	
GnRH antagonists	7 (28%)	6 (24%)	
Flare-up	5 (20%)	6 (24%)	
Duration of stimulation (days)	10.2±1.6	10.3±1.9	0.81
Total dose of FSH (IU)	2373±1010	3009±1294	0.06
Serum estrogens at hCG administration (pg/ml)	2432±1139	1995±1088	0.27
Total number of oocytes retrieved	11.5±5.0	5.5±4.0	<0.001
Number of embryos transferred	2.2±0.5	1.5±0.6	<0.001

Data is reported as mean ± SD or median (Interquartile range) or number (%) as appropriate.

The total number of fibroids in pregnant and non-pregnant women were 46 and 41, respectively. The number of women carrying one, two, three and four fibroids were 14 (56%), 4 (16%), 4 (16%) and 3 (12%) and 15 (60%), 4 (16%), 6 (24%) and 0 (0%), respectively (p = 0.41). They were intramural and subserosal in 23 (50%) and 23 (50%) and in 28 (68%) and 13 (32%) cases, respectively (p = 0.13). The number of lesions at baseline and at second assessment coincided in all women. The mean ± SD diameter of the fibroids was 17±10 and 20±11 mm, respectively (p = 0.18). The mean ± SD diameter of the larger lesion per woman was 20±10 and 22±12 mm, respectively (p = 0.70). The median (IQR) volume of the lesions was 1.6 (0.5–5.5) and 2.6 (0.7–7.5) ml, respectively (p = 0.12). The median (IQR) volume of the larger lesion per woman was 2.7 (0.8–11.4) and 3.9 (0.8–16.0) ml, respectively (p = 0.79).

**Figure 1:**
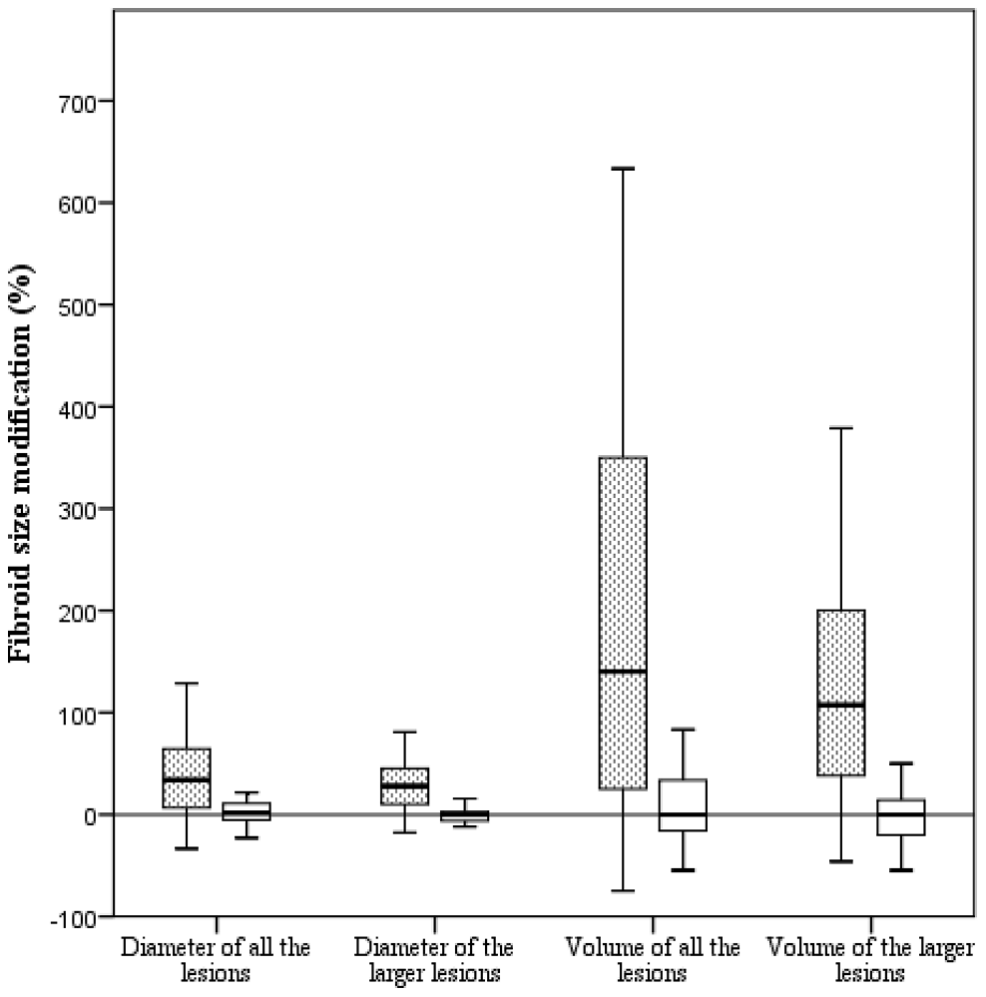
Modification of the dimension of the fibroids. The dotted and white box plots refer to pregnant and non-pregnant women, respectively. All comparisons between fibroids from pregnant and non-pregnant women resulted statistically significant (p<0.001). Statistical tests used were the paired Student *t*-test to compare the diameters and the non-parametric Wilcoxon paired test to compare the volumes.

The modification of the fibroid in pregnant and non-pregnant women is shown in Table 3. A statistically significant increase emerged exclusively in the pregnant group (p<0.001 for all variables). All the data were transformed into percentage of modification in order to allow inter-group comparisons. The median (IQR) modification of the diameter of all the lesions in pregnant and non-pregnant women was +34% (+6%/+65%) and +2% (−6%/+12%), respectively (p<0.001). The median (IQR) modification of the diameter of the larger lesion in pregnant and non-pregnant women was +28% (+6%/+49%) and 0% (−7%/+3%), respectively (p<0.001). The median (IQR) modification of the volume of all the lesions in pregnant and non-pregnant women was +140% (+23%/+357%) and 0% (−18%/+37%), respectively (p<0.001). The median (IQR) modification of the volume of the larger lesion in pregnant and non-pregnant women was +108% (+19%/+214%) and 0% (−22%/+15%), respectively (p<0.001). These results are illustrated in Figure 1.

**Table 3:** Modification of the dimension of the fibroids in the study groups.

	Pregnant women				Non-pregnant women			
	N	Before	After	p	N	Before	After	p
Diameters (mm)								
Total	46	17±10	23±13	<0.001	41	20±11	20±10	0.39
Larger lesion	25	20±10	26±14	<0.001	25	22±12	21±11	0.20
Volumes (mL)								
Total	41	1.6 (0.5–5.5)	5.2 (1.4–14.7)	<0.001	41	2.6 (0.7–7.5)	2.7 (0.8–9.7)	0.68
Larger lesion	25	2.7 (0.8–11.4)	7.8 (2.3–17.4)	<0.001	25	3.9 (0.8–16.0)	4.0 (0.7–14.2)	0.38

Data is reported as mean ± SD for the diameters and as median (Interquartile range) for the volumes.

Comparisons were made using the paired Student *t*-test and the Wilcoxon test for paired data for the diameters and the volumes, respectively.

In order to investigate whether ovarian hyperstimulation played a role in influencing the growth of the fibroids within the group of pregnant women, we performed a correlation between the percentage of volume growth of all lesions and serum AMH, day 3 serum FSH, total dose of gonadotropins administered, duration of stimulation, serum estrogen at the time of hCG administration and the number of oocytes retrieved. The Spearman correlation coefficients were −0.16 (p = 0.42), −0.19 (p = 0.22), −0.01 (p = 0.95), +0.13 (p = 0.40), −0.11 (p = 0.57) and +0.07 (p = 0.65), respectively.

In order to evaluate whether the enlargement of the fibroids was related to the initial dimension of the lesions, we performed a correlation between the percentage of volume growth of all lesions and their initial dimension. The Spearman correlation coefficient was −0.28 (p = 0.009). Finally, we compared the modification of fibroids in twin pregnancies (5 lesions) and singleton pregnancies (41 lesions) and no significant differences emerged (data not shown).

## Discussion

Fibroids dramatically enlarge during initial pregnancy. In this study, we observed that the volume of the fibroids more than double within 6–7 weeks’ gestation. The magnitude of the growth is remarkable and the difference is highly statistically significant. This effect appears particularly evident for smaller lesions. The discrepancy with the few available evidence (Table 1) is presumably related to a type I error and possibly also to inaccuracies in fibroids measurement [3], [5], [6]. In fact, the larger available study included only twelve women [3] and none of these studies was specifically designed to address the growth of fibroids during initial pregnancy. They were actually conceived to observe the modification of these lesions during the course of pregnancy. Women with prepregnant information on the fibroids were included only occasionally and data on the prepregnant dimension of the lesions was presumably collected retrospectively.

Studies supporting a pregnancy-related growth of fibroids generally claimed a crucial role of sex steroids hormone [3], [7], [11], [12]. Estrogen has been considered to be the primary growth promoter of fibroids and there is also convincing evidence that the maintenance and growth of these lesions is progesterone dependent [12]. Noteworthy, selective progesterone-receptor modulators have been shown to effectively shrink fibroid size [13], [14]. However, it is unlikely that sex steroids are the unique actors involved. In fact, estrogen and progesterone raise progressively but consistently during pregnancy reaching a serum concentration of up to 15–20 ng/ml and 120–150 ng/ml in the last trimester, respectively [15], [16] In contrast, fibroids growth does not follow a similar pattern. Albeit available evidence is contrasting, the emerging vision is that fibroid growth during pregnancy is, if any, not linear. De Vivo *et al*. and Rosati *et al*. reported a deceleration of the growth rate during the second part of pregnancy [3], [7]. Lev-Toaff *et al*. observed an increase in size during the second trimester only in small fibroids whereas all lesions decreased in size in the third trimester regardless of their dimension [1]. Moreover, three studies failed to document any modification [2], [4], [6] and Hammoud et al who recruited women late during pregnancy (16–19 weeks) observed a progressive reduction in the dimension of fibroids [4]. Overall, regardless of the precise pattern of change, it can be consistently concluded from the literature that the modification of the fibroids is not directly related to the increase in serum estrogen during pregnancy. Our results are also consistent with a non-exclusive role of sex steroids. We observed a marked increase in size during early pregnancy when estrogens and progesterone are still low and we failed to document any correlation between serum estrogen at the time of hCG administration and the growth of the fibroids.

Sex steroids are not the unique hormones that markedly modify with the advent of pregnancy. A plethora of other hormones and proteins secreted by the fetal, the placental and the maternal compartments markedly rise during early pregnancy [16]. Noteworthy, it cannot be excluded that these substances may also have a synergic effect on fibroid growth. The potential detrimental effects of all these compounds and their combination have not been systematically evaluated. It is beyond the scope of this article to discuss all of them. However, we herein speculate that hCG may be one of the most significant factors involved. The striking enlargement of fibroids mainly during initial pregnancy may indeed be driven by the typical rapid exponential raise in serum hCG and the particular kinetic of its receptor. The hypothesis of an effect of hCG on fibroid growth is actually not novel and there are convincing *in vitro* studies supporting this possibility. The presence of functional LH-hCG receptors on fibroids has been repeatedly demonstrated [17], [18]. Moreover, functional studies showed that hCG increases fibroid cell number both directly [19] and through an autocrine/paracrine effect mediated by PRL secretion [17], [20]. Interestingly, this effect appears to be extremely rapid. Horiuchi *et al*. observed that hCG determines an up to 500% increase in the number of leyomyoma cells after three days of incubation when compared to cells incubated with medium without hCG. Albeit still significant, this effect becomes less evident after 9 days of incubation since at this time the difference dropped to about +200% [21]. It can be stated that, similarly to what occurs *in vivo* in the primate corpus luteum, the LH-hCG receptor in leiomyoma cells requires the exponential growth of hCG to maintain its stimulating effect. It is indeed well-established that primate corpus luteum becomes less sensitive to LH as the luteal lifespan progresses [22]. More robust luteotropic stimulus in the form of exponential rising levels of LH/hCG is required to extend the functional lifespan of the primate corpus luteum in early pregnancy until luteal activities are assumed by the placenta [22]. Future studies evaluating at short intervals (1–2 weeks) fibroids size modifications from embryo implantation to the end of the first trimester and correlating these changes to serum hCG are required to further support our view.

If confirmed, this hypothesis may open also new therapeutic scenarios. Indeed, the critical role of hCG in this context may also explain the rapid growth of fibroids that more frequently occurs in perimenopause [23]. Women in this period of their reproductive life are typically exposed to episodic consistent raises in gonadotropins and fibroids may receive relevant growing stimuli during these LH peaks [24]. This situation is radically different from the post-menopausal period (typically characterized by fibroids regression) when gonadotropins are steadily high and the promoting effect of estrogens is absent. On this basis, one may speculate that a long term administration of estroprogestins in women during premenopause may prevent possible episodic consistent raises in gonadotropins and the consequent growth of leiomyoma. Clinical evidence is however warranted to support this hypothesis.

Some limitations of our study should be recognized. First of all, we included women who underwent IVF and a confounding effect of controlled ovarian hyperstimulation cannot be excluded. However, we deem this limit of mild relevance for several reasons. We previously demonstrated that fibroids were unchanged in women with these lesions who underwent IVF and failed to become pregnant [8]. This observation was confirmed in the present study when the ultrasound assessment was done earlier, i.e. 4–5 weeks after oocytes retrieval rather than 3–9 months later. In this regard, it has however to be recognized that the magnitude of ovarian responsiveness was milder in the control group. This was expected since ovarian reserve and thus responsiveness to hyper-stimulation is a critical factor in influencing the chances of pregnancy. One may argue that the lower response in the control women may explain the lack of a stimulatory effect. At least, two arguments argue against this possibility. Firstly, in non-pregnant women, the size of the lesions remained identical. If ovarian hyper-stimulation does a play a role, at least a trend toward enlargement should have emerged but this did not occur in both the present data and in our previous study [8]. Secondly, and most importantly, in the group of pregnant women, we failed to observe any correlation between fibroids growth and variables of ovarian responsiveness to hyper-stimulation such as in particular serum estrogens at the time of hCG administration. This latter observation is of critical relevance since it tends to rule out also a second possible criticism, i.e. that the observed impressive rapid fibroid growth may be unique to IVF women. Indeed, women in the control group failed to become pregnant and it may be argued that using non-pregnant women as comparators may not fully overcome the confounding effect of ovarian hyper-stimulation. In other words, ovarian hyper-stimulation may influence fibroid growth only if pregnancy occurs. In fact, the hormonal status in terms of estrogens and progesterone levels markedly differ in women achieving pregnancy spontaneously and in those becoming pregnant through IVF. The elevated number of developing follicles results in more corpora lutea and higher circulating estrogens and progesterone levels. The above mentioned lack of any correlation between ovarian response and fibroid growth however argues against this criticism. Moreover, albeit anecdotal, a relevant increase in fibroid size was observed in the unique recruited woman who became pregnant spontaneously before starting the ovarian hyperstimulation. Further evidence in a series of women achieving pregnancy spontaneously is however required for definite generalizability of our findings to women with fibroids achieving pregnancy spontaneously.

A second possible concern is related to the sonographic assessment. Our diagnosis of fibroids lacks histological confirmation and the process of lesions measurement was inevitably exposed to a certain degree of inaccuracy. Again, we do not estimate that this limitation should question the validity of our conclusions for several reasons. The diagnosis of fibroids using ultrasounds is validated in clinical practice [9] and this diagnostic tool is used by all previous studies on fibroids modification during pregnancy [1]–[7]. Moreover, to further reduce the risk of misdiagnosis, all scans were performed by few and experienced physicians and unclear cases as well as women with more than four lesions were excluded. Noteworthy, the number of lesions at baseline and at the time of second assessment coincided in all studied women. Finally, even if some inaccuracies in volume assessment cannot be excluded, the use of blinded operators and the magnitude of the observed differences tend to exclude a critical role of this limitation.

In conclusion, uterine fibroids rapidly growth during initial pregnancy. Physicians should be aware of this possibility and women can be reassured since this change is expected. This results needs however definite confirmation in a population of women achieving pregnancy spontaneously. Further evidence is also required to better draw the precise pattern of fibroids modification during the subsequent prosecution of pregnancy and to assess whether our findings may be of therapeutic interest.
